# Internet-Based Dementia Prevention Intervention (DementiaRisk): Protocol for a Randomized Controlled Trial and Knowledge Translation

**DOI:** 10.2196/64718

**Published:** 2025-01-27

**Authors:** Anthony J Levinson, Stephanie Ayers, Sandra Clark, Rebekah Woodburn, Maureen Dobbins, Dante Duarte, Roland Grad, Nick Kates, Sharon Marr, Doug Oliver, Alexandra Papaioannou, Karen Saperson, Henry Siu, Gillian Strudwick, Richard Sztramko, Sarah Neil-Sztramko

**Affiliations:** 1 McMaster University Hamilton, ON Canada; 2 McGill University Montreal, QC Canada; 3 Centre for Addiction and Mental Health Toronto, ON Canada

**Keywords:** web-based intervention, internet, eHealth, dementia risk, dementia prevention, Alzheimer disease, education and training, clinical trial, knowledge translation, public health, health literacy, e-learning

## Abstract

**Background:**

Research has shown that engaging in a range of healthy lifestyles or behavioral factors can help reduce the risk of developing dementia. Improved knowledge of modifiable risk factors for dementia may help engage people to reduce their risk, with beneficial impacts on individual and public health. Moreover, many guidelines emphasize the importance of providing education and web-based resources for dementia prevention. Internet-based interventions may be effective, but few have been studied rigorously or widely disseminated. We created DementiaRisk, an award-winning, web- and email-based education platform for the public focused on modifiable risk factors, featuring multimedia e-learning and email “microlearning” content, to help raise awareness and improve knowledge of actions to reduce dementia risk.

**Objective:**

This protocol describes a randomized controlled trial to (1) evaluate whether exposure to DementiaRisk changes knowledge of dementia risk factors, intention to engage in risk reduction activities, and health behaviors related to dementia risk reduction and to (2) explore qualitative aspects including participants’ engagement and satisfaction with the intervention and barriers and facilitators to use.

**Methods:**

Using a sequential explanatory mixed methods design, this study conducts a quantitative analysis followed by a qualitative inquiry to evaluate outcomes and feasibility. In total, 485 participants will be recruited on the web and randomly assigned to 2 groups: one accessing DementiaRisk and the other receiving alternative e-learning on mild cognitive impairment. Assessments will be delivered on the web at baseline (T1), at 4 weeks (T2), and at 2 months after the intervention (T3). Knowledge will be assessed using items from the Dementia Knowledge Assessment Scale, intentions to engage in risk reduction activities will be assessed using items in line with current evidence, and health behaviors related to dementia risk reduction will be assessed using items from the Godin-Shephard Leisure Time Physical Activity Questionnaire along with additional questions related to a range of health status domains. Outcomes and feasibility will be assessed using the Information Assessment Method for patients and consumers. A linear mixed effects model will be used to examine the relationship between each outcome score by group and time point.

**Results:**

This study was approved by the Hamilton Integrated Research Ethics Board on August 24, 2022 (project ID 14886) and received funding in February 2023. Recruitment took place from March 28, 2023, to April 28, 2023, with the final participants completing the intervention by August 18, 2023. Analyses and interpretation of data are ongoing.

**Conclusions:**

DementiaRisk is a readily scalable, technology-enhanced solution for dementia prevention education. It has been designed using evidence-based principles of multimedia learning. It has the potential to scale and spread widely using the open internet, so it may be able to reach a wider audience than traditional in-person educational interventions.

**Trial Registration:**

ClinicalTrials.gov NCT05383118; https://clinicaltrials.gov/study/NCT05383118

**International Registered Report Identifier (IRRID):**

DERR1-10.2196/64718

## Introduction

### Background

Developing a better understanding of how dementia can be prevented and sharing information about how Canadian people can reduce their risk of developing dementia or delay its onset are critical to keeping Canadian people healthy and improving their quality of life. The Landmark Study, which developed evidence-based data modeling to demonstrate the impacts of improving risk reduction efforts to delay onset across the population, suggests that a delay of 1 year could result in nearly 500,000 fewer cases of dementia by 2050 [1]. Additionally, if dementia prevention efforts can delay the onset of dementia by 10 years, then over 4 million new cases of dementia could be avoided by 2050 [1]. Web-based learning about the promotion of brain health is a readily scalable, technology-enhanced solution for dementia risk prevention education. Web-based learning has the potential to reach a wider number of audiences with modifiable risk factors for dementia than traditional face-to-face interventions.

While there have been studies of web-based health information on intentions and behavior change, most of those studies have looked at text-based health information rather than internet-based interventions that have incorporated best practices in instructional design for e-learning. Most of the web-based content about modifying dementia risk factors currently is text based, such as static web pages or pamphlets or booklets. Of the few multimedia e-learning courses available, some require a substantial commitment (eg, weeks long), are synchronous (ie, require real-time participation), are associated with a commercial entity or product for sale, or have a cost associated with them. During the design of our intervention, we could not find any instructionally designed, evidence-based, multimedia e-learning content about dementia risk reduction available in Canada in both languages (English and French). Moreover, very few educational interventions related to dementia risk reduction have been rigorously studied using methods such as randomized controlled trials (RCTs).

While multidomain interventions have been shown to be effective [2], our study will complement those types of more intense, in-person components with a view to expanding awareness of modifiable risk factors to the general population. In addition, while text-based information about risk factors is widely available, DementiaRisk [3]—our instructionally designed, multimedia intervention—is designed to share this knowledge broadly in a way that can improve the understanding of Canadian people. Many interventions about dementia are designed specifically for older adults. However, the development of dementia can begin as early as 20 years before symptom onset or diagnosis [4], so we will be targeting the intervention to all Canadian people of 18 years and older of age to reduce the risk of dementia as early as possible.

A recent EKOS dementia public opinion survey highlighted that there are still many important gaps in the knowledge of Canadian people about dementia risk factors, with only 37% of respondents knowing the link to chronic conditions that affect brain health, such as hypertension, heart disease, and diabetes [5]. Moreover, just over one-tenth believe that hearing loss can increase the risk of developing dementia; and many believe that exposure to toxic chemicals is a risk factor, despite lack of evidence [5].

### e-Learning Instructional Design

**Figure 1:**
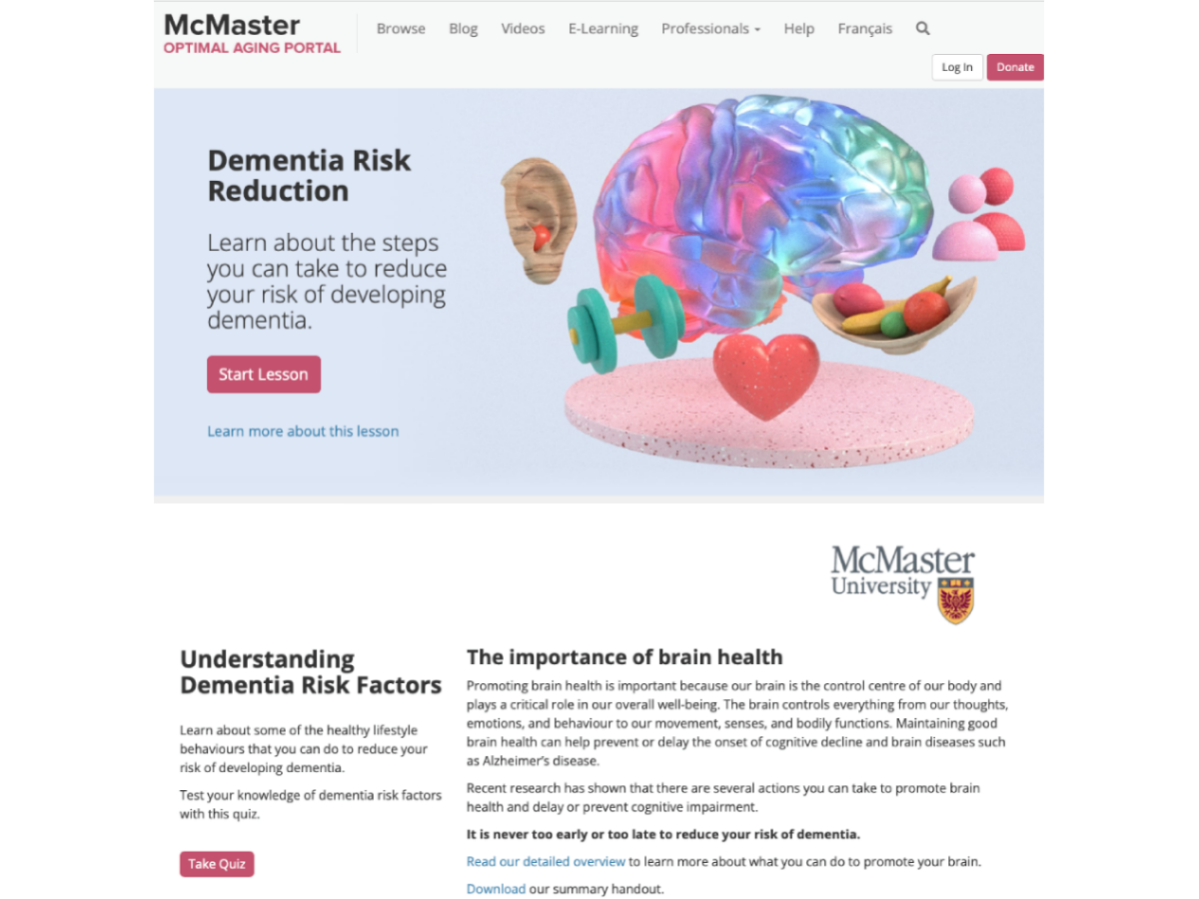
DementiaRisk home page.

DementiaRisk (Figure 1), when compared to other web-based content, is unique because it uses best practices in multimedia e-learning instructional design, is open-access, and includes asynchronous e-learning with email-based microlearning. To our knowledge, it is also the only asynchronous multimedia e-learning dementia education program with French translation available. Key components of the instructional design include the use of the principles of multimedia learning and audio narration; personalization, including the use of a web-based “coach” or embodied instructional agent; segmenting the content into manageable topics to reduce cognitive load; authentic scenarios and worked examples; review questions; and learner control over navigation [6]. An iterative, participatory instructional design and development methodology—the successive approximation method [7]—was used, with extensive involvement and review from a range of experts in dementia care, including care partners.

In this study protocol, we describe a sequential explanatory mixed methods design RCT comparing the efficacy of DementiaRisk, a high-quality, web- and email-based dementia risk reduction education platform that includes asynchronous multimedia e-learning and email-based “microlearning” content, to our alternative web-based learning program on mild cognitive impairment. This study will be an important contribution to the literature on improving knowledge of dementia risk factors through web-based interventions. These innovations are an important complement to traditional approaches to dementia and brain health education, a key component of quality dementia care and public health policy. This is the first research study of the effectiveness of DementiaRisk.

### Research Aim

The overall aim of this study is to explore if and how our dementia risk reduction e-learning influences participants’ knowledge, intentions, and health behaviors related to dementia risk factors.

### Specific Objectives

The specific objectives of this study are (1) to evaluate whether exposure to the e-learning intervention changes knowledge of dementia risk factors, intention to engage in risk reduction activities, and health behaviors related to dementia risk reduction and (2) to explore qualitative aspects such as participants’ engagement and satisfaction with the intervention, as well as barriers and facilitators to use, through surveys.

### Research Questions

This study aims to answer the following research questions: (1) Does DementiaRisk increase knowledge of dementia risk factors? (2) Does DementiaRisk increase intentions to engage in risk reduction activities? (3) Does DementiaRisk increase health behaviors related to dementia risk reduction? and (4) Are participants engaged and satisfied with DementiaRisk?

## Methods

### Study Design

**Figure 2:**
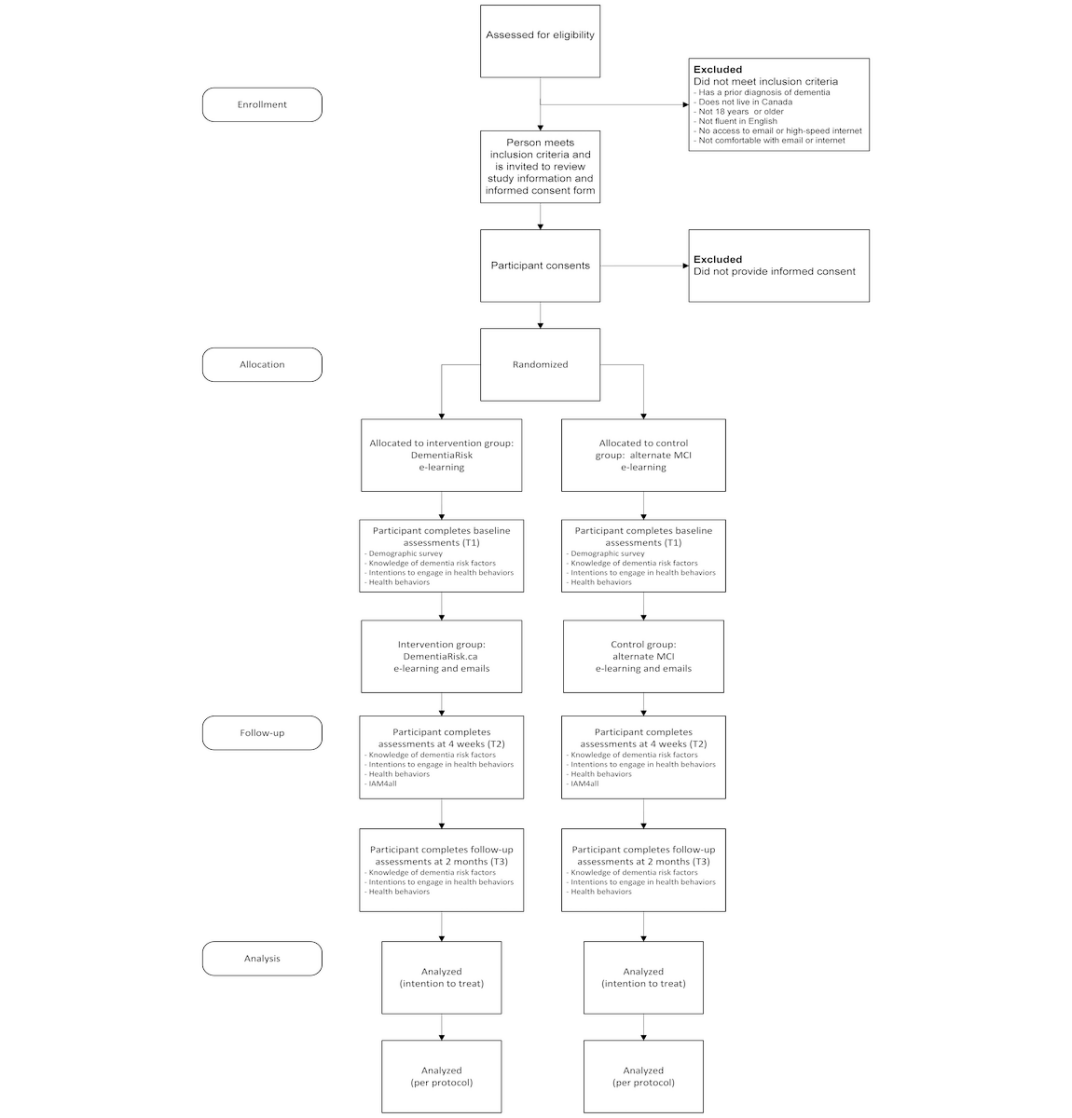
Participant flow through the study. MCI: mild cognitive impairment. For a higher-resolution version of this figure, see Multimedia Appendix 1.

A sequential, explanatory, mixed methods design RCT will be conducted to test the effectiveness of DementiaRisk on increasing knowledge, intentions, and behavior change related to modifiable risk factors of dementia. Participants will receive assessments at the following time points: baseline (T1), at 4 weeks (T2), and at 2 months after the intervention (T3; Figure 2). This specific research design offers a valuable approach because it strategically combines the strengths of both quantitative and qualitative research methods.

The underlying philosophy guiding this mixed methods approach is pragmatism, which emphasizes the practical application of research to address real-world problems [8]. This philosophical approach encourages the use of both quantitative and qualitative methods, allowing for more flexibility from other methodological constraints. This flexibility is particularly advantageous in mixed methods research, as it allows us to adapt our approach to capture unexpected findings that may emerge during the study [9]. By incorporating both quantitative and qualitative data, we can gain a more comprehensive and nuanced understanding of the relationship between DementiaRisk and impacts on knowledge, intentions, and behavior change.

### Participants, Setting, and Procedure

#### Overview

Participants will be recruited using AskingCanadians, a paid panel service that can find representative Canadian participants, including those at increased risk of dementia. AskingCanadians was established in 2005 as a web-based data collection firm, which now allows for access to over 1 million Canadian consumers. The panel will send a copy of our recruitment email to their network where interested participants will be redirected to a survey to identify their eligibility. We will ensure a representative sample of diverse participants with key nonmodifiable and modifiable risk factors for dementia, including a greater percentage of women, some participants with lower level of education, and other risk factors such as hearing impairment, hypertension, smoking, alcohol consumption, and others.

#### Inclusion and Exclusion Criteria

Participants are eligible to participate, if they meet the following self-reported inclusion criteria: (1) they do not have a prior diagnosis of dementia, (2) they reside in Canada, (3) they are 18 years and older of age, (4) they have a good command of the English language, (5) they have access to email and high-speed internet, (6) they are comfortable using email and internet, and (7) they have the ability to grant web-based informed consent. Participants are not eligible to participate if (1) they have a prior diagnosis of dementia, (2) they do not reside in Canada, (3) they are not 18 years and older of age, (4) they do not have a good command of the English language, (5) they do not have access to email and high-speed internet, (6) they are not comfortable using email and internet, and (7) they do not have the ability to grant web-based informed consent. Eligibility screening, informed consent, and surveys will be conducted entirely on the web.

#### Randomization and Allocation Concealment

Participants will be randomized and directed to their assigned group after submitting web-based informed consent using the Division of e-Learning Innovation’s research platform. Participants will be randomized using a permuted block stratified randomization, using education and age as the stratification variables. Our stratified block randomization approach will be based on the following components: (1) level of education: we will stratify participants based on educational background or highest level of education completed: “high school or equivalent,” “some college or university,” and “college or university graduate or graduate degree” and (2) age: we will stratify participants based on ages: “younger than 45 years,” “45-65 years,” and “older than 65 years.”

To ensure a balance between the intervention and the control groups, we will use stratified block randomization with variable block sizes of 4, 6, and 8 (randomly arranged) in a ratio of 1:1. To the best of our ability, efforts will be made to blind participants to their allocation group and study hypotheses. We will ensure that promotional advertisements do not contain logos or direct website links. The informed consent form will not contain the exact outcome measures or the title of the intervention. Allocation concealment will be aided by referring to the intervention as “e-learning related to cognitive impairment and dementia prevention” without biasing participants to study hypotheses or study design.

#### Sample Size Calculation

Using a conservative estimate of a small effect size (0.16, from a meta-analysis of internet health behavior change interventions), with a power of 0.80 and α of .05, we require a total of 388 participants in the study [10,11]. To allow for a 25% dropout rate, we aim to recruit 485 individuals [10,11]. These recruitment numbers and strategies have been used successfully in similar knowledge translation intervention studies and are a feasible target, given the use of the paid panel [12].

### Intervention

#### Overview

**Figure 3:**
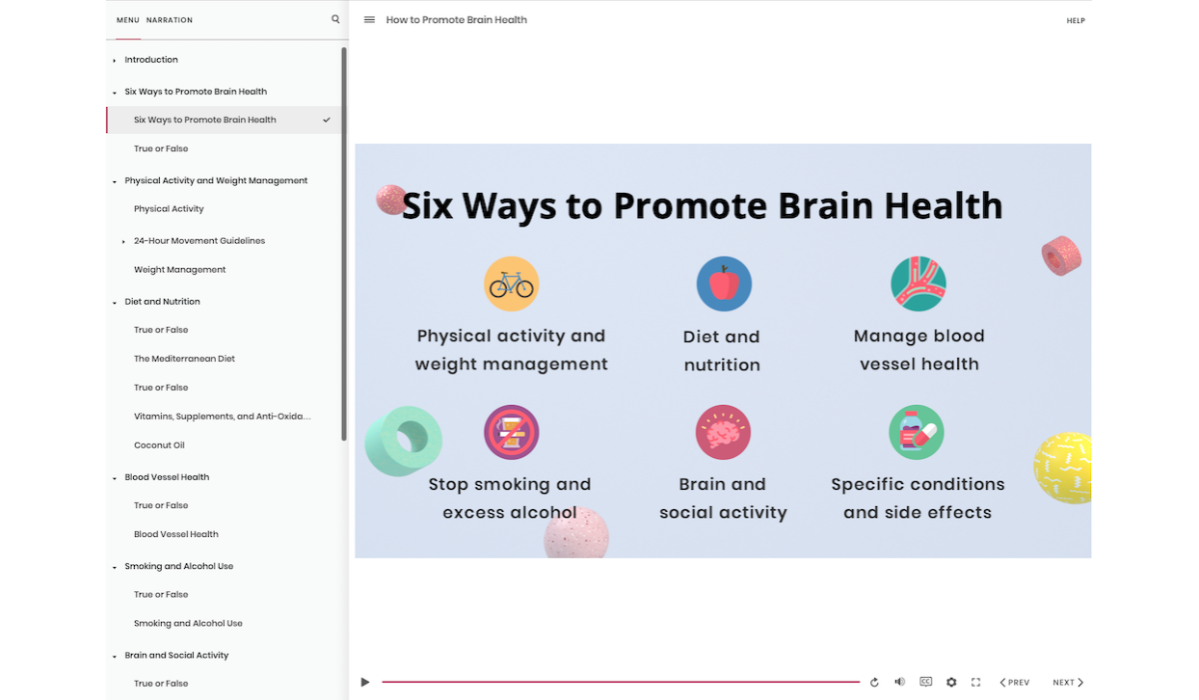
DementiaRisk intervention group e-learning lesson.

Participants in the intervention group will be provided e-learning about dementia risk reduction and promoting brain health (Figure 3), consisting of the following components: (1) one 35-minute multimedia e-learning lesson on promoting brain health and preventing dementia and (2) a series of 12 “microlearning” emails (3 emails per week) with small segments of content to reinforce the material from the lesson. Participant progress is saved, so they can return to the lesson to complete it at another time. Participants will have 4 weeks to complete the intervention.

The lesson will be delivered through the Division of e-Learning Innovation’s learning management system to record participants’ access, progress, and completion of the lessons. This allows us to measure the “dose” of educational exposure to the intervention and to provide automated timed reminders to encourage participants to complete the lesson.

#### Microlearning Emails

In addition to the lessons, participants will also receive 3 microlearning emails per week with a small amount of content that reinforces the information from the lessons. This spaced repetition has been shown to benefit knowledge transfer [6]. Content for the emails will be taken directly from the promoting brain health e-learning lesson.

### Control Condition

Participants randomized to the control group will be provided e-learning about mild cognitive impairment, consisting of the following components: (1) one 20-minute multimedia e-learning lesson on mild cognitive impairment and (2) a series of 12 “microlearning” emails (3 emails per week) with small segments of content to reinforce material from the lesson.

### Outcomes

#### Overview

Primary and secondary outcomes will be assessed at baseline (T1), at 4 weeks (T2), and at 2 months after the intervention (T3). Qualitative data will be collected at 4 weeks (T2; Multimedia Appendix 2). Participants will access web-based surveys through the password-protected research platform hosted by the Division of e-Learning Innovation. Participants will be required to complete all components of the intervention. Surveys will not be submitted unless the participant completes all questions. Participants will not be able to change their answers after submitting and will not be able to submit multiple attempts.

#### Primary Outcome

The primary outcome measure of this study is knowledge change related to dementia risk factors. Knowledge of dementia risk factors will be assessed using a custom 15-item multiple-choice assessment aligned with the content of the intervention as well as the risks and health promotion subdomain of the Dementia Knowledge Assessment Scale [13]. Response options are “false,” “probably false,” “probably true,” “true,” and “I don’t know.” The total maximum score for this assessment is 30 (maximum 2 points per question), with higher scores indicating higher levels of knowledge related to dementia risk factors. Individual scoring is as follows: (1) 2 points for answering “true” to a true question, (2) 2 points for answering “false” to a false question, (3) 1 point for answering “probably true” to a true question, (4) 1 point for answering “probably false” to a false question, (5) 0 points for answering “probably false” or “false” to a true question, (6) 0 points for answering “probably true” or “true” to a false question, (7) 0 points for answering “I don’t know.”

#### Secondary Outcomes

Secondary outcome measures include (1) intentions to engage in health behaviors in line with evidence will be assessed using a 10-item Likert scale–based survey and a composite score and (2) health behaviors related to behavior change for modifying dementia risk factors will be assessed using the Godin-Shephard Leisure Time Physical Activity Questionnaire along with additional questions related to a range of health status and behavior domains including diet, smoking, alcohol consumption, social activity, traumatic brain injury, blood pressure, depression, air pollution, diabetes, sleep habits, cognitive activity, and hearing to determine a composite score. Where possible, these questions were selected from validated tools (Multimedia Appendix 2). Additional data will be collected on engagement with the web-based intervention throughout the study, including (1) e-learning analytics: progress or completion, login activity, and time on the lesson and (2) email campaign analytics: open rate and click-through rate.

#### Qualitative Data

Qualitative data will be collected through structured survey questions and open-ended questions. The effectiveness and impact of the intervention will be assessed using the Information Assessment Method for patients and consumers, a validated questionnaire that assesses outcomes of web-based consumer health information [14]. End-of-intervention and poststudy surveys will include open-ended questions to assess participant satisfaction with the intervention, changes in attitudes, beliefs, and behaviors during the study, and feedback on proposed future dissemination methods.

### Data Analyses

#### Overview

All outcome assessors and data analysts will be blinded to participant allocation. The CONSORT (Consolidated Standards of Reporting Trials) extension for randomized pilot and feasibility trials and CONSORT-EHEALTH [15-17] will guide reporting of the study. The 12-item TIDieR (Template for Intervention Description and Replication) checklist was used for the intervention description [18]. Qualitative reporting will adhere to the COREQ (Consolidated Criteria for Reporting Qualitative Studies) [19].

#### Quantitative Data

All data will be entered into RStudio (version 2022.02.3; Posit PBC) [20-22]. A linear mixed effects model will be used to examine the relationship between total knowledge score and group by time point. The same model will be used to look at health behavior scores and total intention scores. We will look at outcome scores from all 3 time points (baseline [T1], at 4 weeks [T2], and at 2 months after the intervention [T3]). The stratification variables, age and level of education, will be included in all models. Additionally, we will examine possible effect modifiers of age, sex, and education on the relationship between groups and outcomes as well as time point and outcomes. For knowledge scores specifically, we will look at a family history of dementia or having been a care partner of a person living with dementia as a possible effect modifier of the relationship between group and knowledge score and time point and knowledge score. These models will be run for both intention-to-treat and per-protocol participant data.

#### Qualitative Data

All data will be entered into NVivo (version 14; Lumivero) [23]. Data will undergo a conventional, inductive, content analysis approach [24,25]. Data will be systematically examined by a single analyst; codes and themes will be generated based on the content without relying on preconceived theories. To maintain accuracy and credibility, weekly meetings will be held by the analyst and the research team to discuss developing themes, confirm the coding process, and ensure consistency and depth in the representation of the data.

### Ethical Considerations

This study gained Hamilton Integrated Research Ethics Board approval on August 24, 2022 (project ID 14886). Participants were required to provide informed consent and were informed of the length of time of the e-learning, surveys, and email campaign as well as informed about details surrounding data collection, storage, and investigator identities. Participants’ identities and confidentiality were maintained throughout the research study. All participant data were deidentified, and all findings will be nonidentifiable. There is no known risk or harm to participating in this study of publicizing its results or findings. Participants were provided with AskingCanadians points that can be redeemed for various gift cards as compensation for participation in the study.

## Results

The study received funding in February 2023. Recruitment took place from March 28, 2023, to April 28, 2023, with the final participants completing the intervention by August 18, 2023. Analyses and interpretation of data are ongoing. The analysis is expected to be completed by summer 2024, and the results will be published by winter 2024.

## Discussion

### Expected Findings

Many Canadian people are not aware of the potential impact of modifiable risk factors with respect to the development of dementia. DementiaRisk represents an important and innovative contribution to consumer health education in the area of promoting brain health and dementia prevention. Specifically, this study aims to explore the impact of DementiaRisk for reducing modifiable risk factors of dementia, specifically through changes in knowledge of dementia risk factors, intention to engage in risk reduction activities, and health behaviors related to dementia risk reduction. Such improvements could contribute to a reduction in the risk of developing dementia, among other benefits [26]. A range of positive effects has been shown for web-based interventions for various target audiences (ie, health care providers and family or friend care partners) on a wide range of outcomes (eg, knowledge, attitudes, burden, stress, and others) [27-34].

Previously, we have shown that e-learning is effective for health professions’ learning, and we have also outlined some of the more effective instructional design elements with respect to web-based learning [35,36]. Well-designed e-learning that uses best practices in multimedia such as the use of instructional graphics, audio narration, and personalization has been shown to be more effective than e-learning that does not conform to best-evidence instructional design [6,37]. Interventions delivered through the web have the potential to augment traditional face-to-face or paper-based approaches (so-called “blended delivery”); and web-based interventions may allow for greater access to a multitude of users [34,38], facilitating scale and spread [14,39]. DementiaRisk is based on evidence-based studies of dementia risk factors, including addressing several factors at the same time [2]. In addition to a positive impact on participants in the intervention, we anticipate long-term impacts on the Canadian public as well as a benefit for intervention agents (eg, primary care providers and public health units), given the ease of adoption, “trialability,” scale, and spread of DementiaRisk.

### Potential Challenges

We anticipate several limitations in this study. First, we anticipate that due to the nature of self-directed web-based interventions, we may encounter higher dropout rates. To mitigate this, we have opted to use AskingCanadians, a paid panel recruitment service that will allow for ensured efficiency of recruitment, potentially higher retention rates, and the ability to attract a diverse and representative sample of Canadian people. Second, many of the instruments for measuring primary and secondary outcomes were custom-created. Although many of the specific survey questions were pulled from validated tools, there is still the possibility that their novelty may influence result validity, which will require cautious interpretation and the use of qualitative data to enrich findings. Third, although the control group will receive e-learning that is related to mild cognitive impairment, and not dementia risk factors, there is the possibility for some contamination or confounding, with some overlap in content domains and the potential for positive outcomes related to the control group content.

### Conclusions

Canada’s dementia strategy highlights the need to bring awareness to dementia prevention nationally to promote healthy behavior change, in particular, to build the evidence base to inform and promote the adoption of effective risk reduction interventions as well as expand awareness. As the population of older adults living with dementia grows, the demand for high-quality information on dementia prevention is growing as well. Developing a better understanding of how dementia can be prevented and sharing information about how Canadian people can reduce their risk of developing dementia or delay its onset are critical to keeping Canadian people healthy and improving their quality of life. DementiaRisk is a readily scalable, technology-enhanced solution for dementia prevention education. It has the potential to reach a wider number of audiences with modifiable risk factors for dementia than traditional face-to-face interventions.

## Appendix

Multimedia Appendix 1 Participant flow through the study. MCI: mild cognitive impairment.

Multimedia Appendix 2 Outcomes questionnaire questions.

## Abbreviations

CONSORT: Consolidated Standards of Reporting Trials
COREQ: Consolidated Criteria for Reporting Qualitative Studies
RCT: randomized controlled trial
TIDieR: Template for Intervention Description and Replication

## Data Availability

The datasets generated and analyzed during this study can be made available from the corresponding author upon reasonable request.
