# Supplementary figures and images for: Internet-Based Dementia Prevention Intervention (DementiaRisk): Protocol for a Randomized Controlled Trial and Knowledge Translation
Source: JMIR Res Protoc. 2025 Jan 27;14:e64718. doi: 10.2196/64718 (PMC11811655; doi:10.2196/64718)

Enrollment

Allocation

Follow-up

Analysis

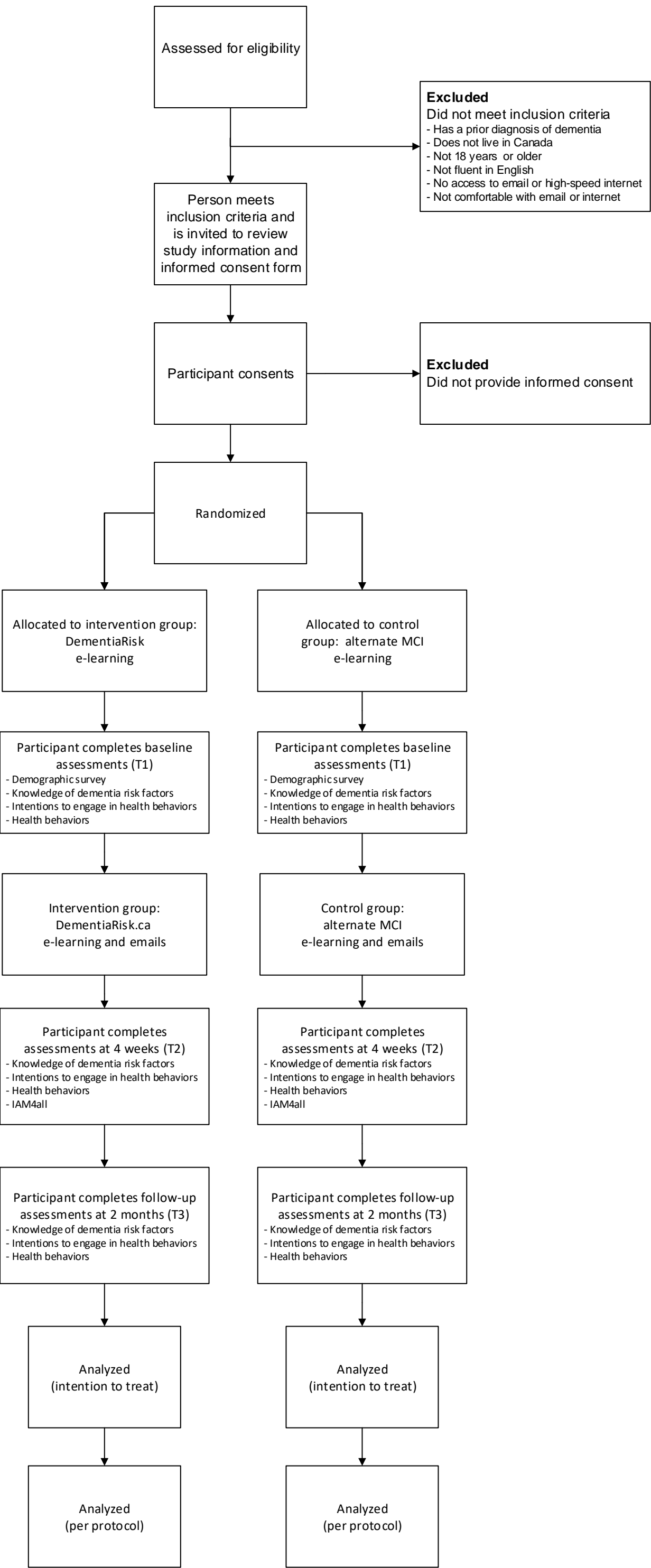

Supplement: Multimedia Appendix 1 [file resprot_v14i1e64718_app1.pdf]
