# Supplementary material for: Internet-Based Dementia Prevention Intervention (DementiaRisk): Protocol for a Randomized Controlled Trial and Knowledge Translation
Source: JMIR Res Protoc. 2025 Jan 27;14:e64718. doi: 10.2196/64718 (PMC11811655; doi:10.2196/64718)
Supplement: Multimedia Appendix 2 [file resprot_v14i1e64718_app2.docx]

| Outcomes | Questions |
| --- | --- |
| Knowledge Assessment | - Maintaining a healthy lifestyle does not reduce the risk of developing the most common forms of dementia. - According to the Canadian 24-Hour Movement Guidelines, adults should participate in 150-minutes of moderate to-vigorous physical activity per week, in bouts of 10-minutes or more. - Maintaining a healthy diet, such as the Mediterranean Diet, has not been shown to promote brain health. - Smoking leads to faster cognitive decline. - Alcohol can cause poor sleep, alter brain function, and increase the risk of developing dementia? - Social isolation can help to maintain cognitive function and reduce the risk of developing dementia. - It is important to maintain good blood vessel health. - Diabetes does not increase your risk for developing dementia. - Getting adequate sleep is not important for brain health. - There is a correlation between the degree or hearing loss and the likelihood of developing dementia. - Traumatic brain injury in midlife does not contribute to developing dementia in later life. - There is no correlation between high cholesterol and increased risk of developing dementia. - Certain medications – both prescribed and over the counter – are associated with increased risk of dementia. - Combining more healthy lifestyle behaviours is associated with a lower risk for developing dementia. - Modifying key lifestyle factors in mid and later life can reduce your risk of developing a dementia by up to 40%. |
| Health Behaviours Assessment | - **Strenuous exercise** (heart beats rapidly) (e.g., running, jogging, hockey, football, soccer, squash, basketball, cross country skiing, judo, roller skating, vigorous swimming, vigorous long distance bicycling)   - Times per week: - **Moderate exercise** (not exhausting) (e.g., fast walking, baseball, tennis, easy bicycling, volleyball, badminton, easy swimming, alpine skiing, popular and folk dancing)   - Times per week: - **Mild/light exercise** (minimal effort) (e.g., yoga, archery, fishing from river bank, bowling, horseshoes, golf, snow-mobiling, easy walking)   - Times per week: - Would you like to increase the amount that you exercise? - Do you follow Canada’s food guide recommendations and eat a variety of healthy foods each day? - How often do you eat a meal that includes meat (beef, pork, lamb, veal, bacon, hamburgers, sausages etc.)? - How often do you eat fruit? - Do you monitor your food intake to reach or maintain a healthy weight? - Do you consider yourself to be:   - Very overweight   - Slightly overweight   - About right   - Slightly underweight   - Very underweight - Please read all the following statements carefully and tick the box next to the one that best describes you.   - I have never smoked a cigarette   - I have only ever tried one or two cigarettes   - I used to smoke sometimes, but I don't now   - I don't smoke cigarettes, but smoke a pipe, cigars, marijuana, or vape   - I smoke cigarettes, but not as many as one per day   - I usually smoke between 1 and 10 cigarettes per day   - I usually smoke between 10 and 20 cigarettes per day   - I usually smoke more than 20 cigarettes per day - Would you like to reduce the amount you smoke? - How many drinks of alcohol do you drink in an average week? - How often in the past 12 months have you had 5 or more drinks on one occasion? - Would you like to reduce the amount that you drink? - How satisfied or dissatisfied are you with your current social life? - In the last 4-weeks, how often did you see your family? - In the last 4-weeks, how often did you see your friends? - Have you ever experienced a head injury (i.e., in a car crash, a fall, a mugging)? - Have you ever lost consciousness after a head injury? - Have you ever had your blood pressure taken? - When was the last time you had your blood pressure taken? - Have you ever been told that you have high blood pressure OR have you ever taken high blood pressure pills? - Have you ever had your cholesterol levels checked? - When was the last time you had your cholesterol levels checked? - Have you ever been told that you have high cholesterol OR have you ever taken medication for your cholesterol levels? - Thinking about your mood over the last 4-weeks, which of the following best describes how you are feeling:   - I am not anxious or depressed   - I am moderately anxious or depressed   - I am extremely anxious or depressed - Have you ever been told that you have depression OR have you ever taken medication for depression? - How would you rate the overall air quality where you live? - To what extent do you think that air pollution is affecting you? - Have you ever had your blood sugar checked? - When was the last time you had your blood sugar checked? - Have you ever been told that you have diabetes or taken medication for diabetes? - Have any of your blood relatives ever been diagnosed with diabetes? - On average, do you feel like you get enough hours of sleep each night? - On average, how many hours of sleep do you get each night? - How satisfied or dissatisfied are you with your current sleep schedule? - In the last 4-weeks, how often did you engage in ‘brain stimulating’ activities? (i.e., reading, video games, crosswords, sudoku, etc.) - Have you ever had your hearing checked? - When was the last time you had your hearing checked? - Have you ever been told that you have hearing loss? - Have you received treatment for hearing loss? - Have any of your blood relatives ever been diagnosed with hearing loss? |
| Health Intentions Assessment | - How likely are you to prioritize engaging with a healthy lifestyle in the next month? - In the next month, how likely are you to engage in 150-minutes of moderate to-vigorous physical activity per week? - How likely are you to engage in consumption of a well-balanced healthy diet (i.e., the Mediterranean diet) in the next month? - How likely are you to reduce your smoking habits in the next month? - How likely are you to reduce your alcohol consumption in the next month? - How likely are you to engage in regular social interactions in the next month? - How likely are you to maintain good blood vessel health in the next month? (i.e., managing high blood pressure, diabetes and high cholesterol; healthy diet and regular physical activity, etc.) - How likely are you to maintain healthy blood glucose (sugar) levels in the next month? (i.e., healthy diet, regular physical activity, etc.) - How likely are you to engage in getting adequate sleep in the next month? - How likely are you to monitor your hearing health in the next month? |
| Feedback Questionnaire | - Was this e-learning lesson relevant? - This e-learning covered a broad range of topics and was not missing any important content or topics. - I was able to complete the e-learning in a reasonable amount of time. - Do you expect any benefit from using this information? - Which benefit(s) are you expecting after taking the e-learning? - I understood the content in this e-learning. - What do you think about this e-learning? - If you answered “other” on the previous question, or there is anything else you would like to tell us about how you expect to benefit from this information, please explain. - Will you use the information from this lesson? - Please tell us how you will use this information. - I would recommend this e-learning to a friend. - Is there an alternative format of learning that you would prefer? - Who would be important for you to tell about this e-learning? (i.e., your doctors, your family, your friends) - Do you have anything else you would like to say about this e-learning or any of the other components? (i.e., the micro-learning campaign, the surveys, the format etc.) |
